# Precisely Tailoring Molecular Structure of Doxorubicin Prodrugs to Enable Stable Nanoassembly, Rapid Activation, and Potent Antitumor Effect

**DOI:** 10.3390/pharmaceutics16121582

**Published:** 2024-12-11

**Authors:** Chengcheng Feng, Yuting Wang, Jiaxu Xu, Yanzi Zheng, Wenhu Zhou, Yuequan Wang, Cong Luo

**Affiliations:** 1 Department of Pharmaceutics, Wuya College of Innovation, Shenyang Pharmaceutical University, Shenyang 110016, China; fengchengcheng5927@aliyun.com (C.F.); wythsj@aliyun.com (Y.W.); xujiaxu201@aliyun.com (J.X.); zhengyanzi1@aliyun.com (Y.Z.); 2 Hunan Key Laboratory of the Research and Development of Novel Pharmaceutical Preparations, Department of Pharmaceutics, School of Pharmaceutical Science, Changsha Medical University, Changsha 410219, China; zhouwenuyaoji@163.com; 3 Department of Pharmaceutics, Xiangya School of Pharmaceutical Sciences, Central South University, Changsha 410013, China; 4 Joint International Research Laboratory of Intelligent Drug Delivery Systems of Ministry of Education, Shenyang Pharmaceutical University, Shenyang 110016, China

**Keywords:** molecular design, doxorubicin prodrug, nanoassembly, drug delivery, drug release

## Abstract

Background: Achieving a balance between stable drug loading/delivery and on-demand drug activation/release at the target sites remains a significant challenge for nanomedicines. Carrier-free prodrug nanoassemblies, which rely on the design of prodrug molecules, offer a promising strategy to optimize both drug delivery efficiency and controlled drug release profiles. Methods: A library of doxorubicin (DOX) prodrugs was created by linking DOX to fatty alcohols of varying chain lengths via a tumor-responsive disulfide bond. In vitro studies assessed the stability and drug release kinetics of the nanoassemblies. In vivo studies evaluated their drug delivery efficiency, tumor accumulation, and antitumor activity in mouse models. Results: In vitro results demonstrated that longer fatty alcohol chains improved the stability of the nanoassemblies but slowed down the disassembly and drug release process. DSSC16 NAs (hexadecanol-modified DOX prodrug) significantly prolonged blood circulation time and enhanced tumor accumulation, with AUC values 14.2-fold higher than DiR Sol. In 4T1 tumor-bearing mouse models, DSSC16 NAs exhibited notably stronger antitumor activity, resulting in a final mean tumor volume of 144.39 ± 36.77 mm^3^, significantly smaller than that of all other groups (*p* < 0.05 by ANOVA at a 95% confidence interval). Conclusions: These findings underscore the critical role of prodrug molecule design in the development of effective prodrug nanoassemblies. The balance between stability and drug release is pivotal for optimizing drug delivery and maximizing therapeutic efficacy.

## 1. Introduction

Cancer remains one of the leading causes of death worldwide, with its incidence and mortality rates continuing to rise [1]. Chemotherapy, as a broad-spectrum antitumor therapy, has long been a cornerstone in the treatment of various cancers [2,3]. Among various chemotherapeutic drugs, doxorubicin (DOX) is a well-established agent with demonstrated efficacy against a broad spectrum of solid tumors, including breast cancer, ovarian cancer, and lymphoma [4]. Its primary mechanism of action involves intercalating into DNA strands and inhibiting the activity of topoisomerase II, ultimately disrupting DNA replication and inducing apoptosis in cancer cells. Despite its potent anticancer activity, the clinical application of DOX is severely restricted due to its dose-dependent cardiotoxicity, which can lead to irreversible heart damage [5,6,7,8]. To address this issue, doxorubicin liposomes (Doxil) have been developed, which encapsulate DOX in lipid vesicles, prolonging its circulation time and reducing direct exposure to the heart. Although nanotechnology helps mitigate the cardiotoxicity of DOX, it does not fundamentally address the inherent nonspecific toxicity of chemotherapy drugs. Doxil still present notable side effects, such as hematological toxicity, oral mucositis, and hand-foot syndrome, which continue to limit their therapeutic efficacy [9,10]. In addition, current DOX-based treatments are limited by several challenges, including the inability to effectively release the drug within the tumor microenvironment.

In response to these challenges, prodrug strategies have emerged as a promising approach to mitigate the toxicity of chemotherapeutic agents. Prodrug is a pharmacologically inactive molecule that undergoes enzymatic or chemical conversion in the body to release the active drug [11,12,13]. This strategy not only improves the physicochemical properties of the drug but reduces toxicity, making it particularly advantageous for chemotherapy [14]. One of the key challenges has been the difficulty in achieving selective drug release at tumor sites due to the heterogeneous nature of tumors. However, the introduction of stimulus-responsive linkers into prodrugs has shown promise in overcoming this issue. For example, tumor cells typically exhibit higher levels of redox stress compared to normal cells, which can be exploited for selective drug activation [15]. By incorporating redox-sensitive linkers, such as disulfide or thioether bonds, prodrugs can be selectively activated within the tumor microenvironment, thereby improving therapeutic efficacy while minimizing side effects [16,17,18]. While this approach offers promising improvements in site-specific drugs activate and reduce nonspecific toxicity, achieving efficient delivery of prodrugs to the tumor site remains a critical challenge for realizing their full therapeutic potential [19,20].

Nanomedicine has revolutionized drug delivery by offering a platform that enhances the therapeutic index of chemotherapeutic agents through targeted delivery and controlled release [21,22]. Nano-drug delivery systems exploit the enhanced permeability and retention (EPR) effect, allowing them to preferentially accumulate in tumor tissues due to the leaky vasculature and poor lymphatic drainage characteristic of tumors [23]. However, traditional nanosystems often suffer from low drug-loading capacity and complex fabrication processes, which limit their clinical application [8]. Interestingly, some small-molecule drugs have demonstrated the ability to self-assemble into carrier-free nanoassemblies without any external materials. These carrier-free nanoassemblies not only maintain the advantages of traditional nanosystems, such as improved drug solubility and tumor accumulation, but also overcome the limitations of low drug loading and complicated preparation [24,25]. Furthermore, by precisely modulating the molecular structure of prodrugs, it is possible to fine-tune their self-assembly behavior and activation kinetics [13,26]. For instance, fatty acids or fatty alcohols, as molecules with good biocompatibility, are often introduced into the design of prodrugs to improve the stability of nanoassembly and increase drug delivery efficiency by changing the intermolecular forces that drive self-assembly [27,28]. Additionally, the incorporation of specific functional groups can modify the physicochemical properties of the prodrug, influencing its activation rate in the tumor microenvironment and ensuring that the drug is released at the optimal therapeutic window [29,30].

**Scheme 1:**
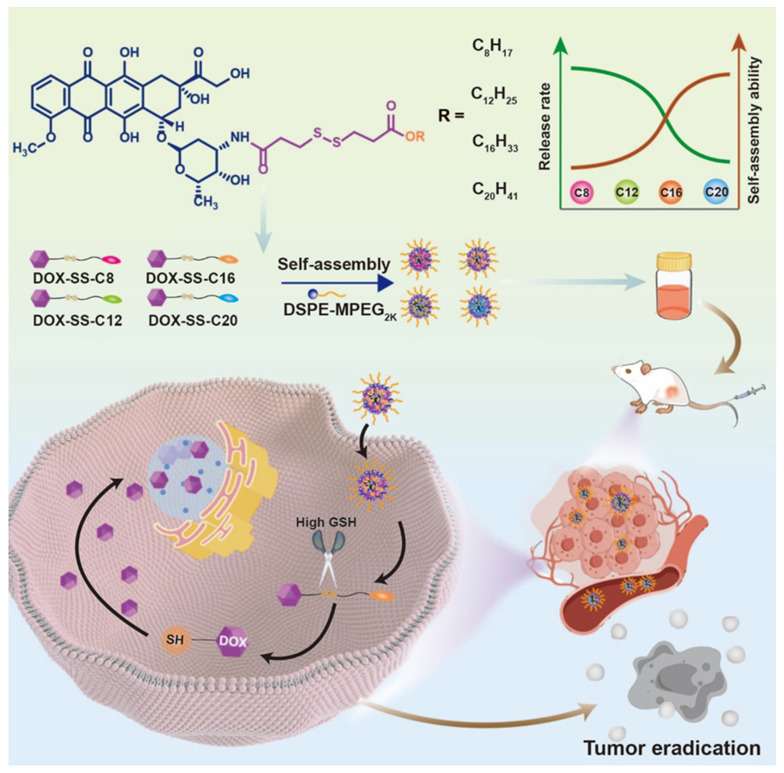
Precisely Programming Prodrug Molecular Structure to Enable Stable Nanoassembly and Rapid Activation.

Building on this concept, a library of DOX-based prodrugs was developed by conjugating DOX to different lengths of fatty alcohols via redox-sensitive disulfide bonds. Interestingly, the self-assembly capacity of the prodrugs increased with the length of the fatty alcohol side chains, leading to the formation of stable nanostructures. However, it was also observed that the disassembly release rate of the nanostructures decreased as the fatty alcohol chain length increased (Scheme 1). Among the various prodrug candidates, the prodrug conjugated with hexadecanol (C16) exhibited the most balanced profile, demonstrating stable nanoparticle formation and efficient drug activation in response to the highly reductive environment in tumor cells. This finding highlights the importance of tuning the balance between prodrug stability and activation kinetics, where intermediate chain lengths offer an optimal combination of prolonged circulation and rapid activation at the tumor site. The ability to fine-tune the self-assembly and activation properties of prodrugs through molecular structure programming offers a powerful tool for the precisely design of next-generation anticancer nano-formulations.

## 2. Materials and Methods

### 2.1. Materials

DOX, EDCI, DMAP, and fatty alcohol were provided by Energy Chemical. MTT and Hoechst 33342 were purchased from Solarbio Science & Technology Co., Ltd. (Beijing, China). DSPE-MPEG_2k_ was obtained from AVT Pharmaceutical Technology Co., Ltd. (Shanghai, China). DiR and C6 were sourced by Meilun Biotechnology Co., Ltd. (Dalian, China). Glass-bottom cell culture dishes, cell culture dishes, and plates were purchased from NEST Biotechnology Co., Ltd. (Wuxi, China).

### 2.2. Synthesis of Fatty Alcohol-DOX Prodrugs

Four disulfide bond-linked fatty alcohol-DOX prodrugs (DSSC8, DSSC12, DSSC16, and DSSC20) were synthesized in three similar steps. First, 3,3′-dithiodipropionic acid (4 mmol) was dissolved in 3 mL of acetic anhydride. The mixture was stirred at 25 °C for 2 h under nitrogen, after which toluene was repeatedly added to dry the solution. Next, the crude product was immediately dissolved in anhydrous dichloromethane. Fatty alcohols (1-octanol, 1-dodecanol, 1-hexadecanol, or 1-eicosanol, 4 mmol) and 4-dimethylaminopyridine (DMAP, 0.4 mmol) were then added, and the reaction mixture was stirred at 25 °C for 12 h under nitrogen. The resulting intermediates were purified using silica gel column chromatography. In the final step, the intermediate (1 mmol), HBTU (3 mmol), and DIPEA (3 mmol) were dissolved in 50 mL of dimethylformamide. After stirring for 2 h in an ice bath under nitrogen, DOX (1 mmol) was added, and the reaction was allowed to proceed at 25 °C for 48 h, with TLC used to monitor completion. The final red solid product was obtained through preparative liquid chromatography with a yield exceeding 60%. The product was confirmed by mass spectrometry and NMR spectroscopy.

### 2.3. Preparation of DOX Prodrug NAs

The four prodrug NAs were prepared through a one-step nanoprecipitation process. The prodrugs (1 mg) and DSPE-MPEG_2k_ (0.25 mg) were dissolved in a 1:1 (*V*/*V*) mixture of absolute ethanol and tetrahydrofuran (0.2 mL), which was then injected into 2 mL of water under stirring. The ethanol and tetrahydrofuran were subsequently removed from the dispersion under reduced pressure at 30 °C.

For the preparation of C6-labeled prodrug nanoassemblies used in cellular uptake studies, a mixed tetrahydrofuran solution of C6, prodrugs, and DSPE-MPEG_2k_ was gradually added into deionized water. The tetrahydrofuran was then removed. A similar method was used to prepare DiR-labeled prodrug nanoassemblies for in vivo biodistribution studies. The particle size of C6-labeled and DiR-labeled prodrug nanoassemblies were measured using a Zetasizer. C6 and DiR are stored at −20 °C before use.

The particle size and polydispersity index (PDI) of the prodrug nanoassemblies were measured using a Zetasizer. We conducted multiple independent repetitions under identical conditions (n = 3). Their morphology was examined by transmission electron microscopy (TEM). TEM samples were prepared by applying a drop of the prodrug nanoassembly solution onto a copper grid. Afterward, 2% phosphotungstic acid was applied to the grid, which was dried thoroughly before imaging.

### 2.4. In Vitro Stability Study

1 mL of the nanoassemblies (0.5 mg/mL) was injected into 10 mL of PBS (pH 7.4) or PBS containing 10% fetal bovine serum (FBS) at 37 °C, respectively. 1 mL of the nanoassemblies (0.5 mg/mL) was injected into 10 mL of PBS (pH 7.4) or PBS containing DTT at 37 °C, respectively. The particle size was assessed using a Zetasizer at specific time intervals. For storage stability, PEGylated prodrug nanoassemblies (0.5 mg/mL) were stored at 4 °C, and particle size was measured at 0, 4, 6, 20, and 24 days.

### 2.5. Nanoassembly Mechanisms

Molecular docking simulations were used to explore the intermolecular forces between prodrug molecules. Firstly, the three-dimensional structures of prodrugs were constructed using an energy minimization approach. Subsequently, molecular docking was performed on the Yinfu Cloud platform (Guangzhou Yinfu Information Technology Co., Ltd., Guangzhou, China) to analyze the driving forces involved in the self-assembly process. Additionally, multiple simulations were conducted. Additionally, NaCl, SDS, and urea were employed to verify the presence of these forces within the prodrug nanoassemblies. In brief, the nanoassemblies were dispersed in NaCl, SDS, and urea in a shaking incubator. Particle size was measured using a Zetasizer (NanoZS, Malvern Co., UK) to assess changes.

### 2.6. Drug Release

The drug release was analyzed using high-performance liquid chromatography (HPLC). Briefly, prodrug nanoassemblies (equivalent to 200 μg of DOX) were added to 30 mL of PBS containing 25% anhydrous ethanol in the presence of either 0 or 1 mM DTT. The mixture was placed in a constant temperature shaker at 37 °C. 0.2 mL of the sample solution was collected and analyzed by HPLC to detect the release of DOX-SH as well as the remaining prodrugs at predetermined time points. We conducted multiple independent repetitions under identical conditions (n = 3).

### 2.7. Cell Culture

The mouse breast tumor cell line (4T1 cells) and mouse prostate cancer cell line (RM-1 cells) were obtained from the cell bank of the Chinese Academy of Sciences (Beijing, China). The 4T1 and RM-1 cells were cultured in RPMI 1640 medium supplemented with 10% FBS, streptomycin (100 μg/mL), and penicillin (100 units/mL). The CT26 and 3T3 cells were cultured in Dulbecco’s Modified Eagle Medium (DMEM) with 10% FBS, streptomycin (100 μg/mL) and penicillin (100 units/mL). All cell lines were maintained in a humidified incubator at 37 °C with 5% CO_2_. Prior to experimental use, FBS was stored at −20 °C, while RPMI 1640 medium and Dulbecco’s Modified Eagle Medium (DMEM) were stored at 4 °C.

### 2.8. Cellular Uptake

To quantitatively assess cellular uptake, 4T1 cells were seeded at a density of 2 × 10^5^ cells per well. The original medium was replaced with fresh medium containing either C6 solution (C6 Sol) or C6-labeled prodrug nanoassemblies (equivalent to 200 ng/mL of C6), and the cells were incubated for either 0.5 or 2 h. After incubation, the cells were washed three times with cold PBS. The cells were then trypsinized, collected, and resuspended in PBS for flow cytometry analysis using flow cytometer (BD FACSCalibur, BD Biosciences, San Jose, CA, USA).

The cellular uptake of the four prodrug nanoassemblies was studied using 4T1 cells. Briefly, 4T1 cells were seeded in 24-well plates preloaded with glass coverslips at a density of 5 × 10^4^ cells/well and cultured for 12 h. The culture medium was then replaced with fresh medium containing C6 solution or C6-labeled prodrug nanoassemblies (equivalent to 200 ng/mL of C6), and the cells were incubated for either 0.5 or 2 h. After incubation, the C6-containing medium was removed, and the cells were washed three times with cold PBS and fixed with 4% formaldehyde. The cell nuclei were stained with Hoechst 33342 for 10 min, and cellular uptake was visualized using confocal laser scanning microscopy (CLSM, C2, Nikon, Tokyo, Japan).

### 2.9. MTT Assay

Cells were seeded at a density of 2 × 10^3^ cells/well. The original culture medium was then replaced with fresh medium containing various concentrations of DOX or the prodrug nanoassemblies and the cells were cultured for an additional 48 h. At the end of the incubation, 20 μL of MTT solution (5 mg/mL) was added to each well, and the plates were incubated for another 4 h at 37 °C. The medium was then replaced with 200 μL of DMSO to dissolve the formazan crystals, and the absorbance at 490 nm was measured using a microplate reader (ThermoFisher Scientific, Waltham, MA, USA). Half-maximal inhibitory concentration (IC_50_) was calculated using GraphPad Prism 8.0.2.

### 2.10. Animal Studies

Sprague-Dawley (SD) rats and BALB/C mice were obtained from the Animal Centre of Shenyang Pharmaceutical University (Shenyang, China). All animal experiments were approved by the Animal Ethics Committee of Shenyang Pharmaceutical University.

### 2.11. Pharmacokinetics

SD rats were randomly divided into five groups (n = 5). Each group received an intravenous injection of either DiR Sol or DiR-labeled prodrug nanoassemblies (2 mg/kg, DiR equivalent) via the tail vein. Blood samples were collected from the ophthalmic vein, and plasma was isolated. Plasma DiR concentrations were quantified using a microplate reader.

### 2.12. Biodistribution

4T1 cells were collected, resuspended in PBS (pH 7.4), and the suspension was injected subcutaneously into the right flank of female BALB/C mice. Once the tumor volume reached approximately 400 mm^3^, the mice were divided into five groups (n = 3) and injected with either DiR Sol or DiR-labeled prodrug nanoassemblies (2 mg/kg, DiR equivalent). Fluorescence imaging was performed using a in vivo imaging system (PerkinElmer IVIS Spectrum, Waltham, MA, USA). Mice were sacrificed at the peak fluorescence time points, and tumors and major organs (heart, liver, spleen, lung, and kidney) were harvested for fluorescence imaging.

### 2.13. In Vivo Antitumor Efficacy

When the average tumor volume reached approximately 90 mm^3^, the mice were randomly divided into six groups (n = 5): Control (PBS), DOX solution (DOX Sol), DSSC8 NAs, DSSC12 NAs, DSSC16 NAs, and DSSC20 NAs. Each formulation was administered every other day for a total of five doses at an equivalent DOX concentration (10 mg/kg). Tumor volume and body weight were measured daily. Two days after the final treatment, blood samples were collected, and the mice were sacrificed. The major organs and tumor tissues were photographed and fixed with 4% paraformaldehyde for H&E and TUNEL staining.

### 2.14. Statistical Analysis

All data were calculated using GraphPad Prism 8.0.2 and presented as the mean value ± SD. Statistical significance was determined using Student’s *t*-test (two-tailed) or one-way ANOVA. Significance was set at *p* < 0.05, with * *p* < 0.05, ** *p* < 0.01, and *** *p* < 0.001.

## 3. Results and Discussion

### 3.1. Synthesis of Fatty Alcohol-DOX Prodrugs

In order to investigate the effect of prodrug molecular structure on nanoassembly and drug activation, four DOX prodrugs were synthesized by linking four fatty alcohols of varying chain lengths (C8, C12, C16, and C20) to DOX via disulfide bonds. These prodrugs were named DSSC8, DSSC12, DSSC16, and DSSC20, respectively. The synthesis routes for the four prodrugs were illustrated in Figure S1, and the chemical structures of the final products were confirmed via high-resolution mass spectrometry (HRMS) and 1H NMR (Figures S2–S5).

### 3.2. Nanoassembly Capacity of DOX Prodrugs

**Figure 1:**
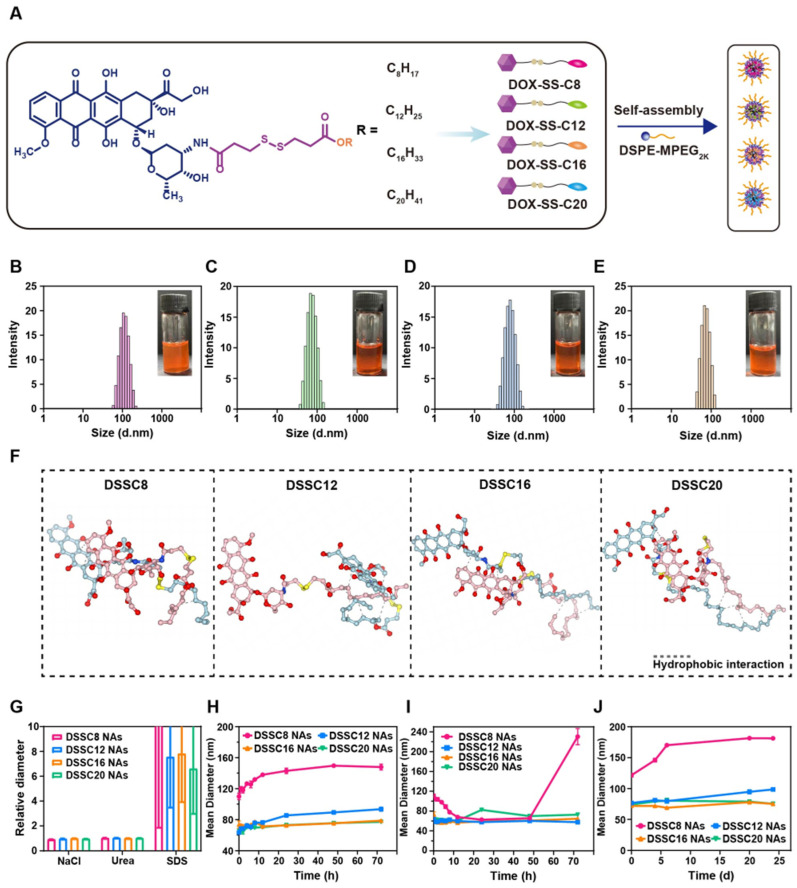
Preparation and characterization of fatty alcohol-DOX prodrug nanoassemblies. (**A**) Schematic diagram of prodrug nanoassemblies; Photographs and particle size distribution profiles of (**B**) DSSC8 NAs, (**C**) DSSC12 NAs, (**D**) DSSC16 Nas, and (**E**) DSSC20 NAs; (**F**) Molecular docking simulation of prodrug nanoassemblies pink and light blue: Carbon atom, red: Oxygen atom, yellow: Sulfur atom, blue: Nitrogen atom; (**G**) The size change curves of prodrug nanoassemblies (n = 3); Colloidal stability of prodrug nanoassemblies incubated in (**H**) PBS (pH 7.4) and (**I**) PBS (pH 7.4) containing 10% FBS (n = 3); and (**J**) Long-term colloidal stability of prodrug nanoassemblies at 4 °C (n = 3).

In our previous study, non-PEGylated prodrug nanoassemblies exhibited low stability under simulated physiological conditions, resulting in suboptimal in vivo pharmacokinetic behavior. To address this issue, PEGylation is commonly applied as a solution [31]. As illustrated in Figure 1A, four PEGylated DOX prodrug nanoassemblies (DSSC8 NAs, DSSC12 NAs, DSSC16 NAs, and DSSC20 NAs) were prepared using a one-step nanoprecipitation method. The majority of the nanoassemblies (DSSC12 NAs, DSSC16 NAs, and DSSC20 NAs) exhibited particle sizes in the range of 60–70 nm, whereas DSSC8 NAs had a larger particle size of approximately 100 nm (Figure 1B–E and Table S1). TEM analysis confirmed that all the nanoassemblies possessed uniform spherical nanostructures (Figure S6). These results suggest that the DOX prodrugs modified with longer fatty alcohol chains (C12, C16, and C20) had stronger self-assembly abilities compared to that with shorter fatty alcohol chain (C8).

To further explore the molecular mechanism behind how fatty alcohol chain length affects the nanoassembly capacity of the prodrugs, molecular simulations were performed. As shown in Figure 1F, hydrophobic forces were identified as the dominant driving mechanism for the nanoassembly process. Molecular force disruption experiments further supported these findings: while the particle sizes of all prodrug nanoassemblies increased significantly within 2 h when incubated in PBS (pH 7.4) containing SDS, no substantial changes were observed in PBS containing NaCl or urea (Figure 1G). These results confirmed that hydrophobic forces played a critical role in the assembly process, in alignment with the molecular simulation outcomes. As the hydrophobicity of the prodrugs increased with the length of the fatty alcohol chains, the nanoassembly capacity improved, which explains the observed trend: DSSC8 NAs < DSSC12 NAs < DSSC16 NAs < DSSC20 NAs in terms of assembly strength.

The zeta potentials of the prodrug nanoassemblies ranged between −20 and −30 mV (Table S1), supporting their colloidal stability and helping to prevent particle aggregation. To assess the stability of these nanoassemblies in physiological environments, we monitored their particle size changes in PBS (pH 7.4) and PBS containing 10% FBS. DSSC8 NAs exhibited significant particle size variations under both conditions, while DSSC12 NAs showed moderate increases in size in PBS (pH 7.4). In contrast, DSSC16 NAs and DSSC20 NAs demonstrated excellent colloidal stability, with minimal changes in particle size in both PBS and PBS containing 10% FBS (Figure 1H,I). These results were further corroborated by storage stability assessments (Figure 1J). To simulate the stability of prodrug nanoassemblies in the tumor microenvironment, we monitored the changes in particle size of the prodrug nanoassemblies in PBS (pH 7.4) or PBS containing DTT at predetermined time points. The results demonstrated that all the prodrug nanoassemblies were responsive to the tumor-reducing microenvironment, exhibiting varying degrees of particle size increase. The extent of this increase followed the order: DSSC8 NAs > DSSC12 NAs > DSSC16 NAs > DSSC20 NAs (Figure S7).

### 3.3. In Vitro Prodrug Activation

**Figure 2:**
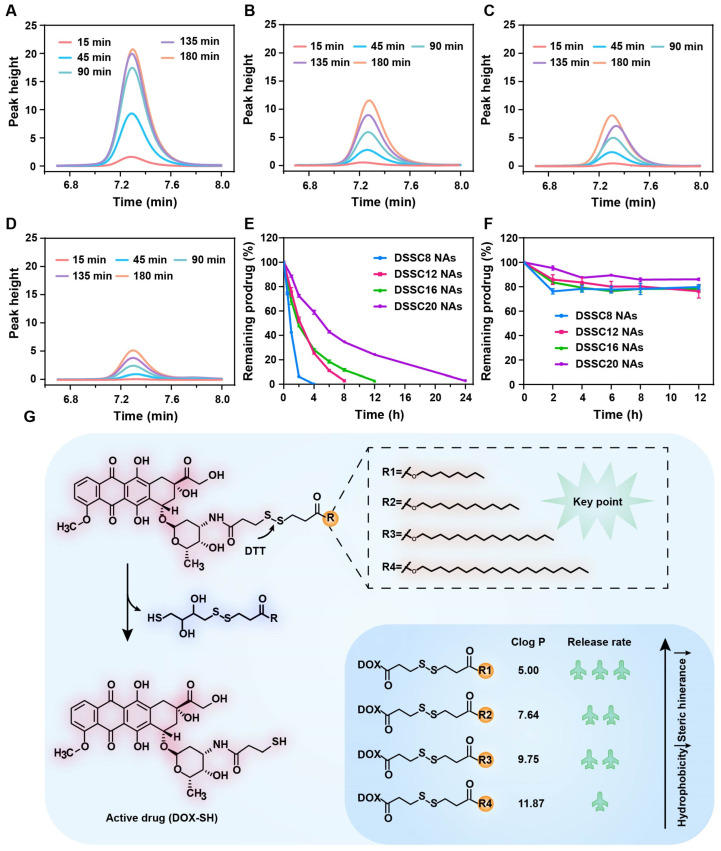
DTT-triggered prodrug activation and mechanism. The in vitro drug release of the active intermediate (DOX-SH) at 5 mM DTT from DSSC8 NAs (**A**), DSSC12 NAs (**B**), DSSC16 NAs (**C**), and DSSC20 NAs (**D**) (n = 3). The proportion of remaining prodrug in 1 mM (**E**) and 0 mM (**F**) (n = 3); (**G**) DTT-triggered drug release mechanism of DSSC8 NAs, DSSC12 NAs, DSSC16 Nas, and DSSC20 NAs.

Tumor cells are characterized by a highly reductive microenvironment, which distinguishes them from normal cells. To exploit this, we incorporated reduction-sensitive disulfide bonds into our prodrugs, enabling their targeted release at tumor-specific sites and minimizing toxic side effects on surrounding healthy tissues. We simulated the tumor microenvironment using DTT to examine the reduction-responsive activation of the prodrug nanoassemblies. Unlike the ester bond-based prodrugs, which release the parent drug through reduction, the DOX prodrugs with amide bonds did not release the parent drug directly due to their stability in PBS. Instead, the active intermediate (DOX-SH) was released. As shown in Figure 2A–D, the four prodrug nanoassemblies released active intermediates within 3 h in the following order: DSSC8 NAs > DSSC12 NAs > DSSC16 NAs > DSSC20 NAs. To further quantify the efficiency of this reduction-responsive release, we analyzed the prodrugs’ residual curves. DSSC8 NAs were almost entirely degraded within 4 h, while DSSC20 NAs took approximately 24 h to degrade. Additionally, DSSC12 NAs exhibited a slightly faster degradation rate compared to DSSC16 NAs (Figure 2E). Overall, the release rate decreased as the fatty alcohol chain length increased.

We then delved deeper into the drug release mechanisms. Disulfide bonds were attacked by DTT, to break into sulfhydryl intermediates. As shown in Figure 2G, DTT reduces the prodrug molecules to DOX-SH by cleaving the disulfide bond. We hypothesized that the varying lengths of aliphatic alcohol chains, which modify the hydrophobicity and steric hindrance of the DOX prodrugs, could affect their susceptibility to DTT. Calculated Log P values for DSSC8, DSSC12, DSSC16, and DSSC20 NAs were 5.00, 7.64, 9.75, and 11.87, respectively. DSSC8 NAs, with the lowest hydrophobicity, demonstrated the fastest release rate. In contrast, DSSC20 Nas, with the highest hydrophobicity, significantly slowed the DTT-mediated cleavage of sulfur atoms, thereby delaying the release of DOX-SH. Notably, in the absence of DTT, the prodrugs of DSSC8, DSSC12, DSSC16, and DSSC20 remained largely stable, confirming their good stability under normal physiological conditions (Figure 2F).

### 3.4. Cellular Uptake

**Figure 3:**
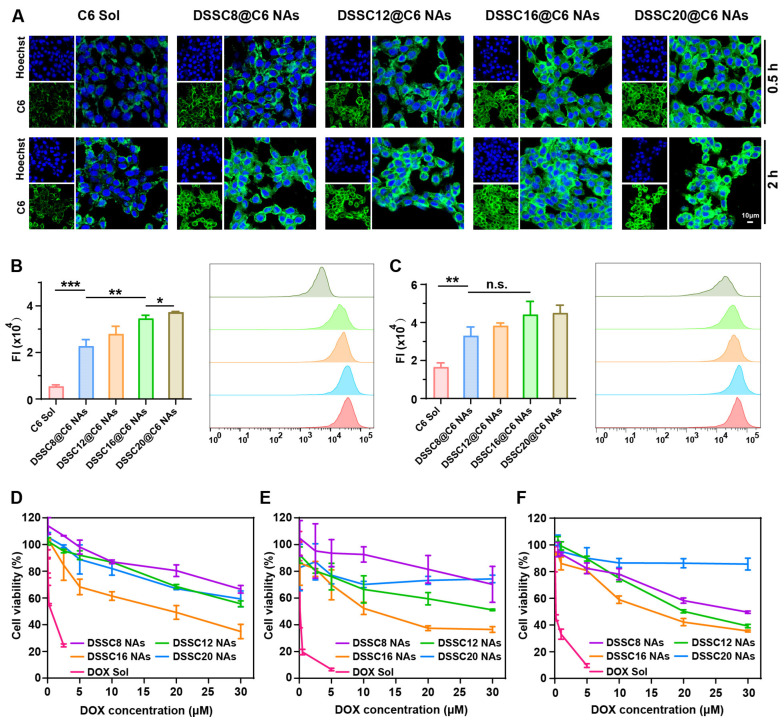
Cellular uptake and MTT assay. (**A**) CLSM images of 4T1 cells incubated with C6 Sol or C6-labeled prodrug-nanoassemblies for 0.5 h and 2 h (scale bar = 10 μm); Flow cytometric analyses of 4T1 cells incubated with C6 sol or C6-labeled prodrug-nanoassemblies for (**B**) 0.5 and (**C**) 2 h (n = 3) * *p* < 0.05, ** *p* < 0.01, *** *p* < 0.001, n.s. no significant; Cell viability after treated with various concentrations of DOX Sol and prodrug nanoassemblies for 48 h in (**D**) 4T1 cells, (**E**) RM-1 cells, and (**F**) CT26 cells (n = 3).

For antitumor drug delivery, the drug can only inhibit tumor growth once they are taken up by tumor cells. To investigate this, we examined the cellular uptake of the prodrug nanoassemblies. First, we measured the fluorescence spectra of DOX solution, the prodrugs, and the prodrug nanoassemblies, revealing distinct fluorescence intensities among them (Figure S8). To avoid variations in fluorescence intensity due to drug release, we introduced C6 as a fluorescent probe to accurately quantify cellular uptake and the addition of C6 had minimal impact on the size and potential of the prodrug nanoparticles (Table S2). In brief, 4T1 cells were incubated with C6 Sol or C6-labeled prodrug nanoassemblies (DSSC8@C6 NAs, DSSC12@C6 NAs, DSSC16@C6 NAs, and DSSC20@C6 NAs) for 0.5 and 2 h. As shown in Figure 3A–C, the intracellular fluorescence intensity of the C6-labeled nanoassemblies was significantly higher than that of the C6 Sol, indicating that the prodrug nanoassemblies achieved much greater cellular uptake efficiency compared to the free drug. Importantly, the cellular uptake efficiency of the prodrug nanoassemblies was positively correlated with their colloidal stability. Compared to DSSC16@C6 NAs and DSSC20@C6 NAs, the uptake efficiencies of DSSC8@C6 NAs and DSSC12@C6 NAs (particularly DSSC8@C6 NAs) were lower, likely due to their poor stability under physiological conditions.

### 3.5. MTT Assay

Tumor cells typically exhibit elevated levels of reactive oxygen species (ROS) due to enhanced metabolic activity and mitochondrial dysfunction. To counteract the heightened ROS levels, these cells often upregulate their antioxidant defense systems (such as GSH). The disulfide bond in the prodrug is cleaved under the reductive conditions present in tumor cells, resulting in the release of DOX-SH. This mechanism effectively differentiates the toxicity between tumor cells and normal cells, enhancing the therapeutic selectivity. Therefore, the inhibitory effect of DOX Sol and prodrug nanoassemblies on cells was evaluated by MTT assay in three tumor cell lines (4T1, RM-1, and CT26) and one normal cell line (3T3). As shown in Figure 3D–F and Figure S9, and Table S3, cells treated with prodrug nanoassemblies exhibited weaker inhibition compared to cells treated with DOX Sol, likely due to the gradual drug release. Notably, DOX Sol significantly inhibited 3T3 cells, whereas the prodrug nanoassemblies, designed for tumor-specific activation, showed no significant inhibitory effect in 3T3 cells, underscoring their enhanced biosafety. The differences among the prodrug nanoassemblies across tumor cell lines were likely influenced by the heterogeneity of the tumor redox microenvironments, which affected drug release rates. DSSC8 NAs exhibited the worst inhibitory effect on tumor cells, followed by DSSC12 NAs. Despite the rapid drug release rate of DSSC8 NAs, their poor nanoassembly capacity and stability led to subpar results. Conversely, DSSC20 NAs, characterized by superior stability, demonstrated reduced toxicity effects due to slower drug release. In contrast, DSSC16 NAs achieved the most effective inhibitory effect in tumor cells, striking an optimal balance between colloidal stability, efficient cellular uptake, and moderate drug release behavior. MTT assay has several inherent limitations, including non-specificity, susceptibility to interference, and reliance on mitochondrial activity as a proxy for overall cell viability. To obtain a more accurate and comprehensive understanding of cellular viability, it is import to use alternative methods such as the clonogenic assays, apoptosis assays, and live/dead cell staining. However, it must be acknowledged that the MTT assay, as a convenient and rapid method, has been widely used in anticancer drug research [32,33,34,35].

### 3.6. Pharmacokinetics and Biodistribution

**Figure 4:**
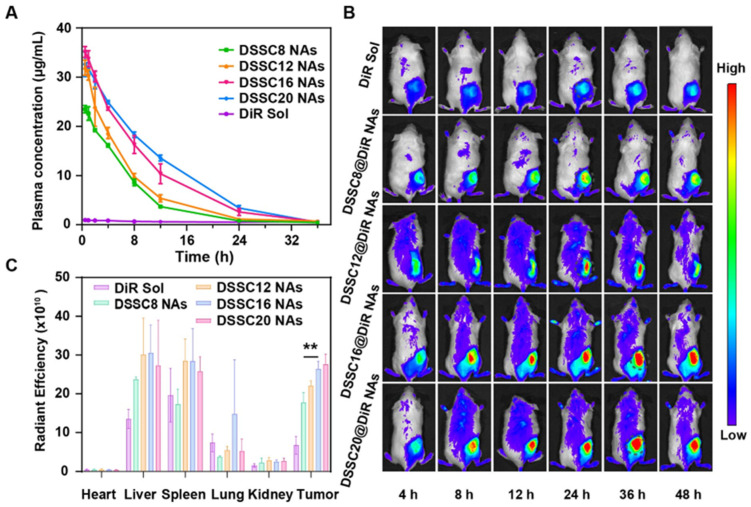
Pharmacokinetic and in vivo biodistribution. (**A**) Pharmacokinetic profiles of DiR Sol and DiR-labeled prodrug-nanoassemblies following a single intravenous administration of 2 mg/kg (DiR equivalent) (n = 5); (**B**) Living images of 4T1 tumor-bearing BALB/c mice treated with DiR Sol and DiR-labeled prodrug-nanoassemblies at a DiR equivalent dose of 1.5 mg/kg; (**C**) Quantitative analysis of excised tissues treated with various formulations at the time when tumor accumulation was brightest (n = 3). ** *p* < 0.01.

As previously discussed, the chain length of fatty alcohols can influence the in vitro stability and drug release behavior of prodrug nanoassemblies. After transvascular administration, the in vivo delivery fate of these nanoassemblies becomes even more critical, affecting their circulation time and accumulation at the tumor site. A large number of studies have shown that the blood circulation time of nanoassemblies is positively correlated with the colloidal stability. Better colloidal stability often predicts higher in vivo delivery efficiency. Therefore, we next examined how different fatty alcohol chain lengths impact the pharmacokinetic behavior and tissue distribution of the prodrug nanoassemblies in vivo. First, we evaluated the pharmacokinetic profiles of DiR Sol and four DiR-labeled prodrug nanoassemblies in healthy rats. As shown in Figure 4A, DiR Sol was rapidly cleared from the bloodstream after intravenous injection, indicating that the free drug did not effectively accumulate in tumor tissues. In contrast, the four DiR-labeled prodrug nanoassemblies exhibited improved pharmacokinetics, with extended blood circulation times. More importantly, based on the area under the concentration-time curve (AUC) results, the fatty alcohol chain length significantly influenced the in vivo pharmacokinetics of the prodrug nanoassemblies (Table S4). DSSC16 NAs and DSSC20 NAs showed a marked increase in AUC, with values 14.2-fold and 16.1-fold higher than DiR Sol, respectively. This may be attributed to the excellent assembly capacity and favorable colloidal stability under physiological conditions. In comparison, DSSC8 NAs and DSSC12 NAs showed more modest increases of 8.4-fold and 10.3-fold, likely due to their poorer self-assembly and colloidal stability, resulting in less favorable pharmacokinetic behavior.

Next, we assessed the in vivo biodistribution of the prodrug nanoassemblies by tracking the fluorescence of DiR in 4T1 tumor-bearing BLAB/C mice. As shown in Figure 4B,C and Figure S10, the fluorescence intensity at the tumor site was significantly higher for the prodrug nanoassemblies compared to DiR Sol. DSSC16 NAs and DSSC20 NAs reached maximum tumor accumulation at 36 h and exhibited stronger fluorescence signals, attributable to their superior colloidal stability and prolonged circulation time. In contrast, DSSC8 NAs and DSSC12 NAs displayed lower tumor accumulation. These results demonstrated that the length of the fatty alcohol chains affected the nanoassembly ability and colloidal stability of the prodrug nanoassemblies, which in turn influences in vivo delivery and pharmacokinetics. Improved nanoassembly and stability contribute to better pharmacokinetic behavior and higher tumor-targeted accumulation.

### 3.7. In Vivo Antitumor Efficacy

**Figure 5:**
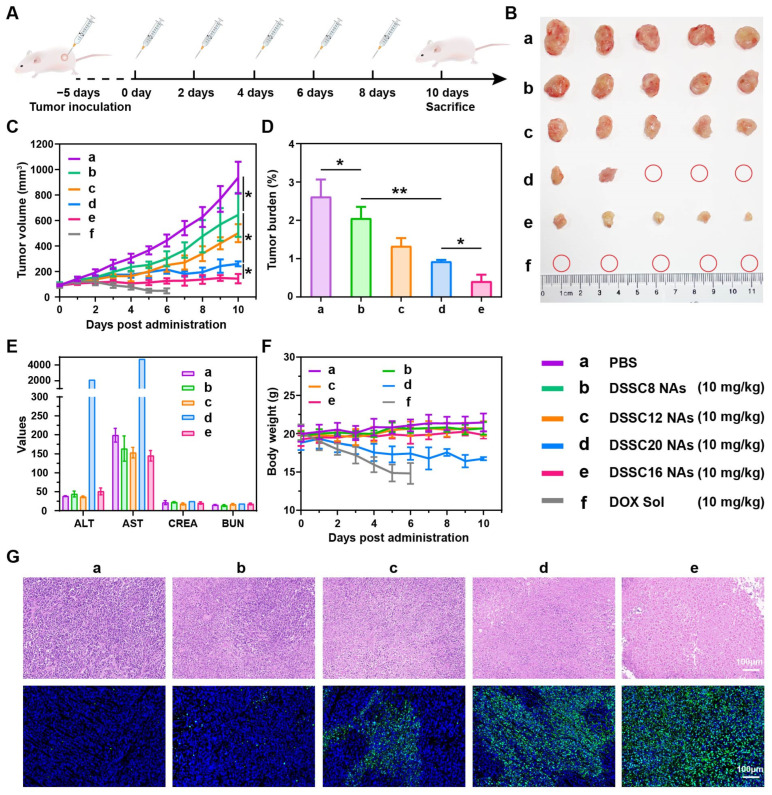
In vivo antitumor efficacy of prodrug nanoassemblies. (**A**) Treatment schedule; (**B**) Digital images of excised tumors from 4T1 tumor-bearing BALB/c mice following various treatments (× represents the death of the mice); (**C**) Tumor growth curves post-treatment; (**D**) Tumor burden following different treatments; (**E**) Hepatic and renal function assessments post-treatment, including alanine aminotransferase (ALT, U/L), aspartate aminotransferase (AST, U/L), creatinine (CREA, μmol/L), and blood urea nitrogen (BUN, mg/dL) (n = 3). (**F**) Body weight changes during treatment; (**G**) H&E and TUNEL staining of tumor tissues after treatment (Scale bar = 100 μm). * *p* < 0.05, ** *p* < 0.01.

Finally, we established a 4T1 tumor-bearing mouse model to assess the in vivo antitumor effects of the prodrug nanoassemblies (Figure 5A). As shown in Figure 5B–D, both DOX Sol and the four prodrug nanoassemblies demonstrated tumor growth inhibition to varying degrees compared with the saline group. However, due to the severe toxicity of DOX Sol on normal tissues, particularly the heart, mice in this group began to die after the second administration, with all mice succumbing by day 6. In contrast, the prodrug nanoassemblies significantly mitigated damage to normal tissues due to their tumor-specific drug release. Surprisingly, mice treated with DSSC20 NAs also experienced high mortality, with only two mice surviving by day 10. Previous studies have reported that as the carbon chain length of fatty alcohols increases, their metabolites can cause harm to the body. DSSC8 NAs and DSSC12 NAs showed weaker antitumor efficacy, likely due to lower colloidal stability, suboptimal pharmacokinetics, and insufficient tumor accumulation. DSSC16 NAs exhibited the strongest antitumor effects. While DSSC20 NAs demonstrated good colloidal stability and high tumor accumulation, their antitumor activity was inferior to that of DSSC16 NAs, possibly due to slower drug release within tumor cells. Moreover, mice treated with DSSC20 NAs showed a significant decrease in body weight and abnormal liver and kidney function markers, suggesting that the metabolism of the C20 fatty alcohol may cause damage to these organs and compromised biosafety (Figure 5E,F). H&E staining and TUNEL assay results further confirmed that DSSC16 NAs displayed the most potent antitumor activity, likely due to their excellent colloidal stability, efficient prodrug activation, strong cellular uptake, prolonged blood circulation, and superior tumor-specific accumulation (Figure 5G). Additionally, as shown in Figure 5F and Figure S11, body weight remained stable after treatment, and no significant histological damage was observed in H&E-stained sections of major organs (heart, liver, spleen, lungs, and kidneys), indicating that DSSC16 NAs are promising candidates as potent antitumor agents.

## 4. Conclusions and Discussion

In conclusion, our research underscores the critical role of precisely programming prodrug molecular structures in achieving a delicate balance between stable nanoassembly and rapid activation. This approach addresses a key challenge in drug delivery systems: how to maintain drug stability in circulation while ensuring rapid and effective drug release once the drug reaches the tumor microenvironment. The exploration of various fatty alcohol chain lengths linked to DOX via tumor-responsive disulfide bonds revealed that longer chains enhance the stability of nanoassemblies while slowing down disassembly and drug release. This delayed release may not always meet the dynamic demands of targeted therapy, particularly in the rapidly changing tumor microenvironment, where prompt drug release is essential for effective treatment. Notably, the hexadecanol-modified DOX prodrug (DSSC16) emerged as the most effective formulation, achieving optimal stability and rapid drug activation, leading to significant antitumor efficacy in 4T1 tumor-bearing mouse models. These findings illuminate the intricate relationship between nanoassembly stability, drug release kinetics, and therapeutic outcomes, providing a valuable framework for the rational design of prodrug-based nano-drug delivery systems to enhance antitumor efficacy.

While fatty alcohol-modified prodrugs demonstrated significant potential in improving pharmacokinetics and therapeutic efficacy, certain limitations warrant critical consideration. For example, DSSC20, which features a longer fatty alcohol chain, exhibited severe biosafety concerns, including high mortality rates and potential liver and kidney toxicity in vivo. These adverse effects are likely attributable to the metabolic byproducts of the longer fatty alcohol chains, which can accumulate and cause organ damage. Moreover, the variability in fatty alcohol metabolism across individuals may pose challenges for clinical translation, potentially leading to unpredictable toxicity profiles.

## Data Availability

The original contributions presented in the study are included in the article/Supplementary Material, further inquiries can be directed to the corresponding author/s.

## Supplementary Materials

The following supporting information can be downloaded at https://www.mdpi.com/article/10.3390/pharmaceutics16121582/s1; Figure S1: The synthetic routes of disulfide bond-linked fatty alcohol–DOX prodrugs; Figure S2: (a) MS of DSSC8 prodrug. (b) ^1^H NMR of DSSC8 prodrug; Figure S3: (a) MS of DSSC12 prodrug. (b) ^1^H NMR of DSSC12 prodrug; Figure S4: (a) MS of DSSC16 prodrug. (b) 1H NMR of DSSC16 prodrug; Figure S5: (a) MS of DSSC20 prodrug. (b) ^1^H NMR of DSSC20 prodrug; Figure S6: The transmission electron microscope image of DSSC8 NAs, DSSC12 NAs, DSSC16 Nas, and DSSC20 NAs. Scale bar represents 200 nm; Figure S7: The size change of prodrug nanoassemblies in PBS without (A) or with (B) DTT (n = 3); Figure S8: Fluorescence spectrum of DOX Sol, prodrug Sol, and prodrug nanoassemblies at a DOX concentration of 2 μg/mL; Figure S9: Cell viability after treated with various concentrations of DOX Sol and prodrug nanoassemblies for 48 h in 3T3 cells; Figure S10: Ex vivo fluorescent imaging of 4T1 tumor-bearing BALB/c mice treated with DiR Sol and DiR-labeled prodrug-nanoassemblies at the time when tumor accumulation was brightest; Figure S11: H&E staining images of the major organs of mice bearing 4T1 tumor xenografts after the last treatments (Scale bar = 100 μm); Table S1: The characteristics of PEGylated prodrug nanoassemblies (n = 3); Table S2: The characteristics of C6-labeled prodrug nanoassemblies (n = 3); Table S3: IC_50_ values (μmol/L) of DOX Sol and DOX prodrugs nanoassemblies against three tumor cell lines and 3T3 cell (n = 3); Table S4: Pharmacokinetic parameters of DiR Sol and DiR-labeled prodrug nanoassemblies (n = 5).
