# Supplementary material for: Precisely Tailoring Molecular Structure of Doxorubicin Prodrugs to Enable Stable Nanoassembly, Rapid Activation, and Potent Antitumor Effect
Source: Pharmaceutics. 2024 Dec 11;16(12):1582. doi: 10.3390/pharmaceutics16121582 (PMC11679313; doi:10.3390/pharmaceutics16121582)
Supplement: Supplementary file 1 [file pharmaceutics-16-01582-s001.zip › pharmaceutics-3322734-supplementary.pdf]

pharmaceutics

Article

Supplementary Materials: Precisely Tailoring Molecular Structure of Doxorubicin Prodrugs to Enable Stable Nanoassembly, Rapid Activation, and Potent Antitumor Effect

Chengcheng Feng, Yuting Wang, Jiaxu Xu, Yanzi Zheng, Wenhu Zhou, Yuequan Wang and Cong Luo

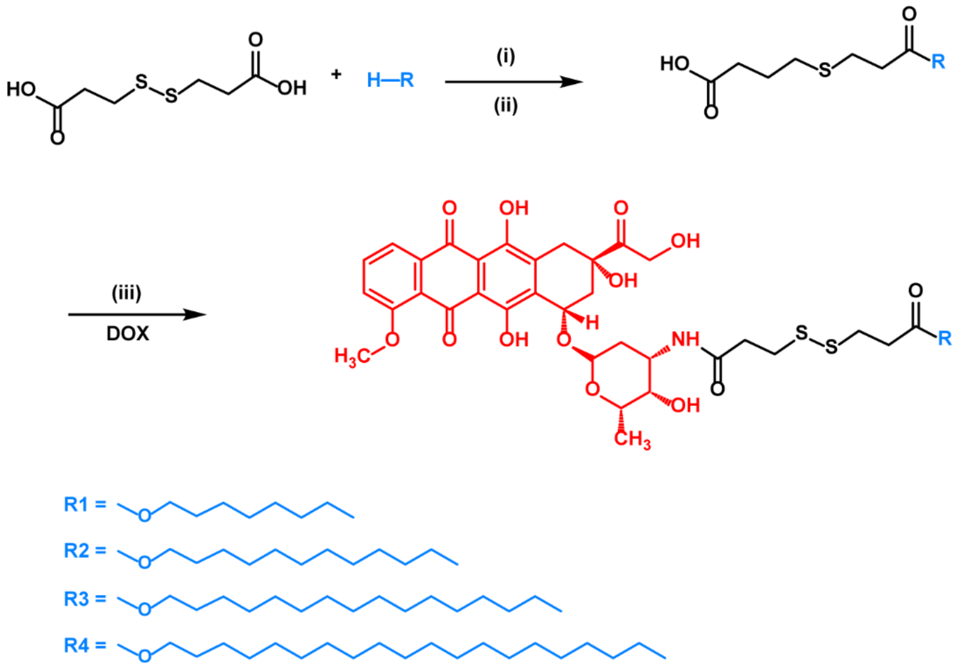

Figure S1. The synthetic routes of disulfide bond-linked fatty alcohol-DOX prodrugs. (**i**) acetic anhydride, r.t.;(**ii**) DMAP, r.t.;(**iii**) HBTU, DIPEA , r.t. Different structures are represented by different colors.

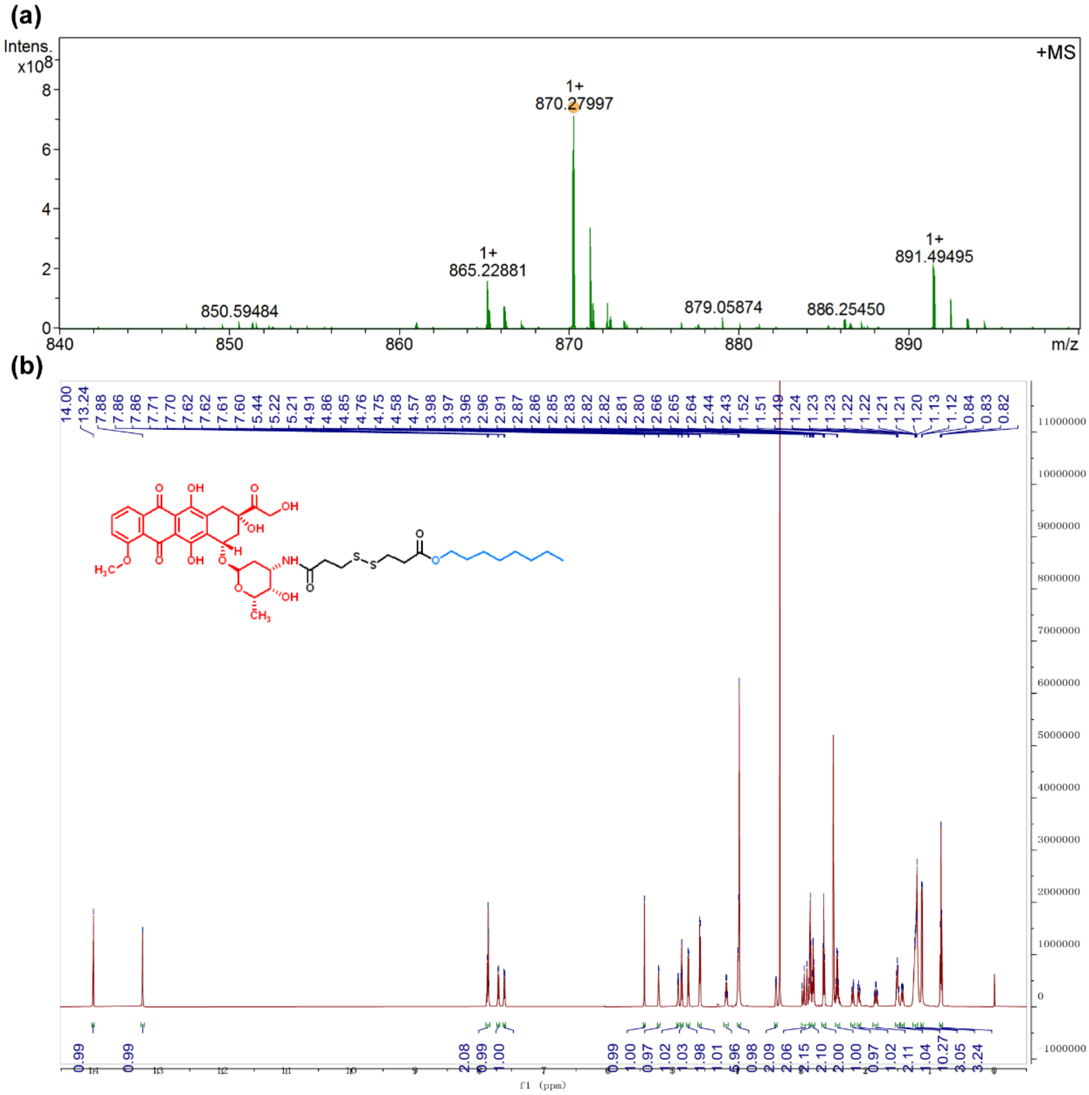

Figure S2. (**a**) MS of DSSC8 prodrug. $[M+Na]^+ = 870.27997$. (**b**) ^{1}H NMR of DSSC8 prodrug. ^{1}H NMR (600 MHz, DMSO-d_6) δ 14.00 (s, 1H), 13.24 (s, 1H), 7.90–7.84 (m, 2H), 7.71 (d, J = 8.1 Hz, 1H), 7.61 (dd, J = 7.7, 2.2 Hz, 1H), 5.44 (s, 1H), 5.21 (d, J = 3.9 Hz, 1H), 4.91 (dd, J = 5.6, 3.4 Hz, 1H), 4.86 (t, J = 6.0 Hz, 1H), 4.75 (d, J = 5.9 Hz, 1H), 4.57 (d, J = 6.0 Hz, 2H), 4.16 (q, J = 6.6 Hz, 1H), 4.01–3.95 (m, 6H), 3.39 (dd, J = 6.3, 2.6 Hz, 1H), 2.99–2.88 (m, 2H), 2.86 (t, J = 6.8 Hz, 2H), 2.83–2.79 (m, 2H), 2.65 (t, J = 6.8 Hz, 2H), 2.44 (q, J = 7.6 Hz, 2H), 2.23–2.17 (m, 1H), 2.10 (dd, J = 14.3, 5.7 Hz, 1H), 1.84 (td, J = 12.9, 3.9 Hz, 1H), 1.51 (p, J = 6.7 Hz, 2H), 1.43 (dd, J = 12.2, 4.5 Hz, 1H), 1.25–1.19 (m, 10H), 1.12 (d, J = 6.5 Hz, 3H), 0.83 (t, J = 7.0 Hz, 3H). The yellow dots represent the molecular ion peaks of the target product.

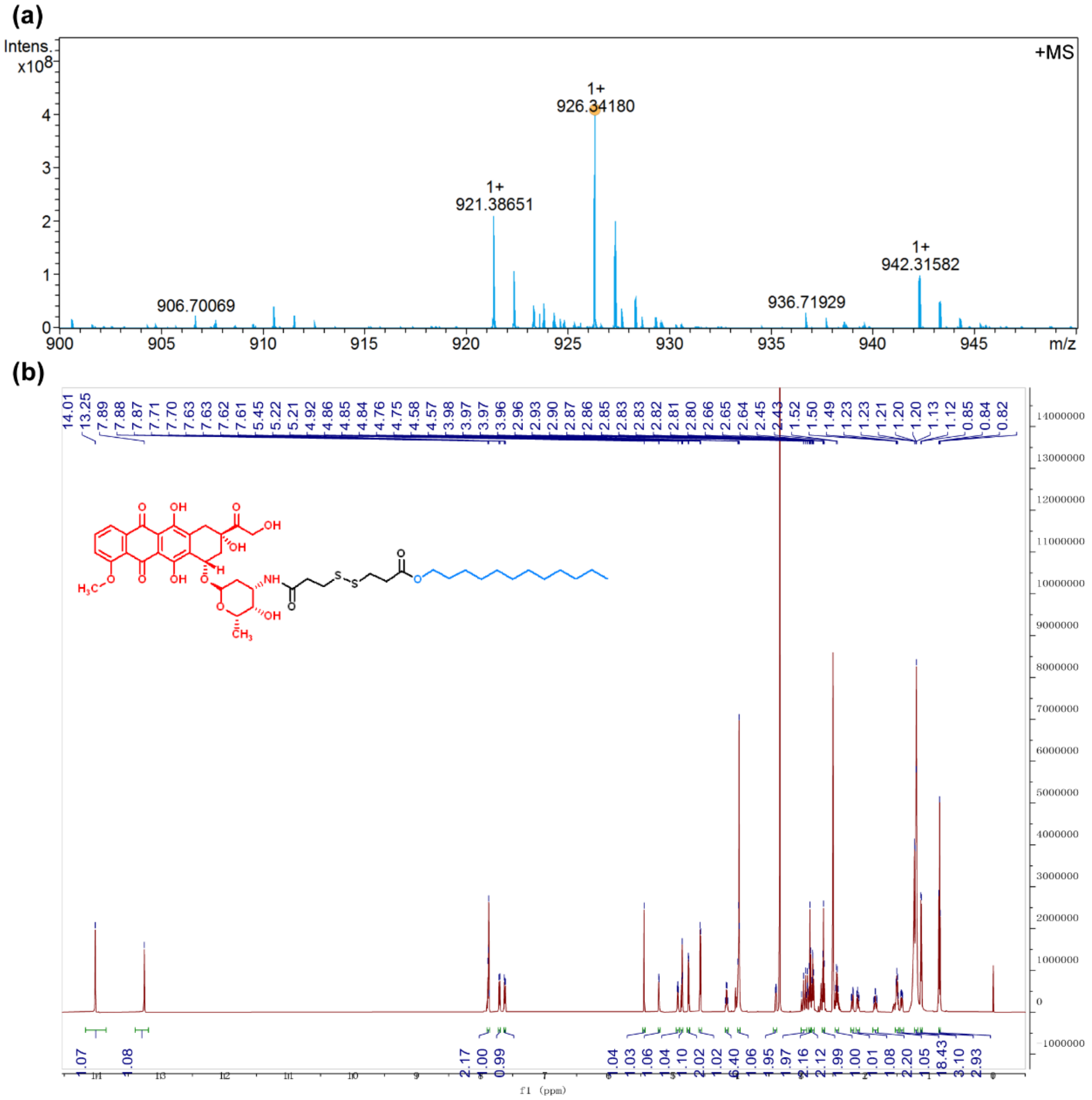

Figure S3. (**a**) MS of DSSC12 prodrug. $[M+Na]^+ = 926.34180$. (**b**) ^1H NMR of DSSC12 prodrug. ^1H NMR (600 MHz, DMSO-d_6) δ 14.01 (s, 1H), 13.25 (s, 1H), 7.91–7.85 (m, 2H), 7.71 (d, *J* = 8.1 Hz, 1H), 7.62 (dd, *J* = 7.0, 2.8 Hz, 1H), 5.45 (s, 1H), 5.22 (d, *J* = 3.9 Hz, 1H), 4.92 (dd, *J* = 5.6, 3.4 Hz, 1H), 4.85 (t, *J* = 6.0 Hz, 1H), 4.75 (d, *J* = 6.0 Hz, 1H), 4.57 (d, *J* = 5.9 Hz, 2H), 4.16 (q, *J* = 6.6 Hz, 1H), 4.02–3.95 (m, 6H), 3.39 (dd, *J* = 6.5, 2.6 Hz, 1H), 3.00–2.90 (m, 2H), 2.86 (t, *J* = 6.9 Hz, 2H), 2.84–2.79 (m, 2H), 2.65 (t, *J* = 6.8 Hz, 2H), 2.47–2.39 (m, 2H), 2.20 (dd, *J* = 14.8, 3.1 Hz, 1H), 2.11 (dd, *J* = 14.2, 5.7 Hz, 1H), 1.84 (td, *J* = 12.9, 3.9 Hz, 1H), 1.50 (q, *J* = 6.9 Hz, 2H), 1.43 (dd, *J* = 12.0, 4.4 Hz, 1H), 1.22–1.18 (m, 18H), 1.12 (d, *J* = 6.3 Hz, 3H), 0.84 (t, *J* = 6.9 Hz, 3H). The yellow dots represent the molecular ion peaks of the target product.

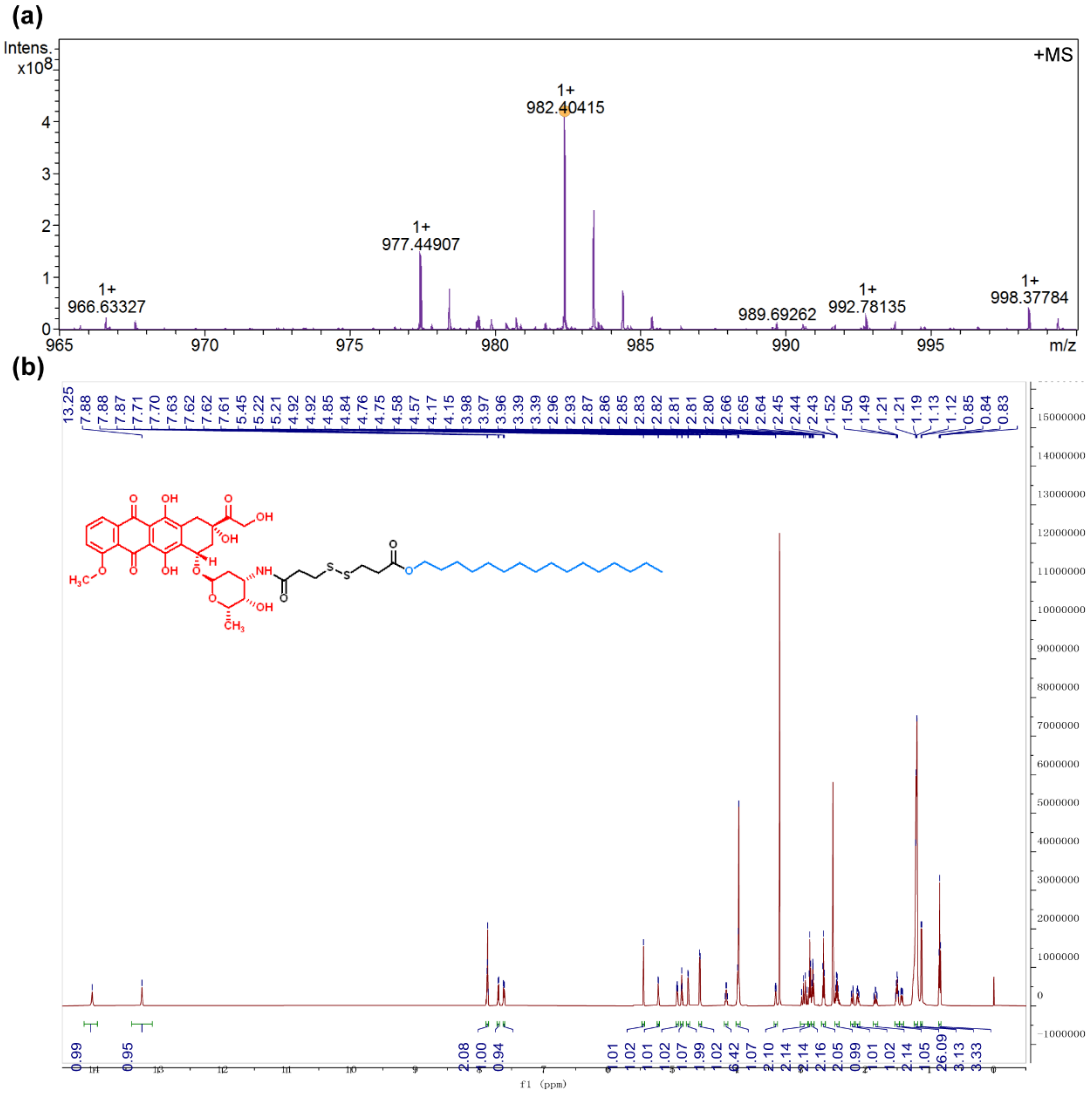

Figure S4. (**a**) MS of DSSC16 prodrug. $[M+Na]^+$ = 982.40415. (**b**) ^1H NMR of DSSC16 prodrug. ^1H NMR (600 MHz, DMSO-d_6) δ 14.02 (s, 1H), 13.25 (s, 1H), 7.91–7.85 (m, 2H), 7.70 (d, *J* = 8.2 Hz, 1H), 7.62 (dd, *J* = 6.8, 3.0 Hz, 1H), 5.45 (s, 1H), 5.22 (d, *J* = 3.8 Hz, 1H), 4.92 (dd, *J* = 5.6, 3.4 Hz, 1H), 4.85 (t, *J* = 5.9 Hz, 1H), 4.75 (d, *J* = 5.9 Hz, 1H), 4.57 (d, *J* = 5.8 Hz, 2H), 4.16 (q, *J* = 6.6 Hz, 1H), 4.01–3.95 (m, 6H), 3.39 (dd, *J* = 6.4, 2.6 Hz, 1H), 2.94 (q, *J* = 18.0 Hz, 2H), 2.86 (t, *J* = 6.9 Hz, 2H), 2.84–2.79 (m, 2H), 2.65 (t, *J* = 6.9 Hz, 2H), 2.49–2.39 (m, 2H), 2.20 (dd, *J* = 14.4, 3.5 Hz, 1H), 2.11 (dd, *J* = 14.2, 5.8 Hz, 1H), 1.84 (td, *J* = 13.0, 4.0 Hz, 1H), 1.50 (p, *J* = 6.7 Hz, 2H), 1.43 (dd, *J* = 12.5, 4.6 Hz, 1H), 1.22–1.18 (m, 26H), 1.12 (d, *J* = 6.6 Hz, 3H), 0.84 (t, *J* = 6.9 Hz, 3H). The yellow dots represent the molecular ion peaks of the target product.

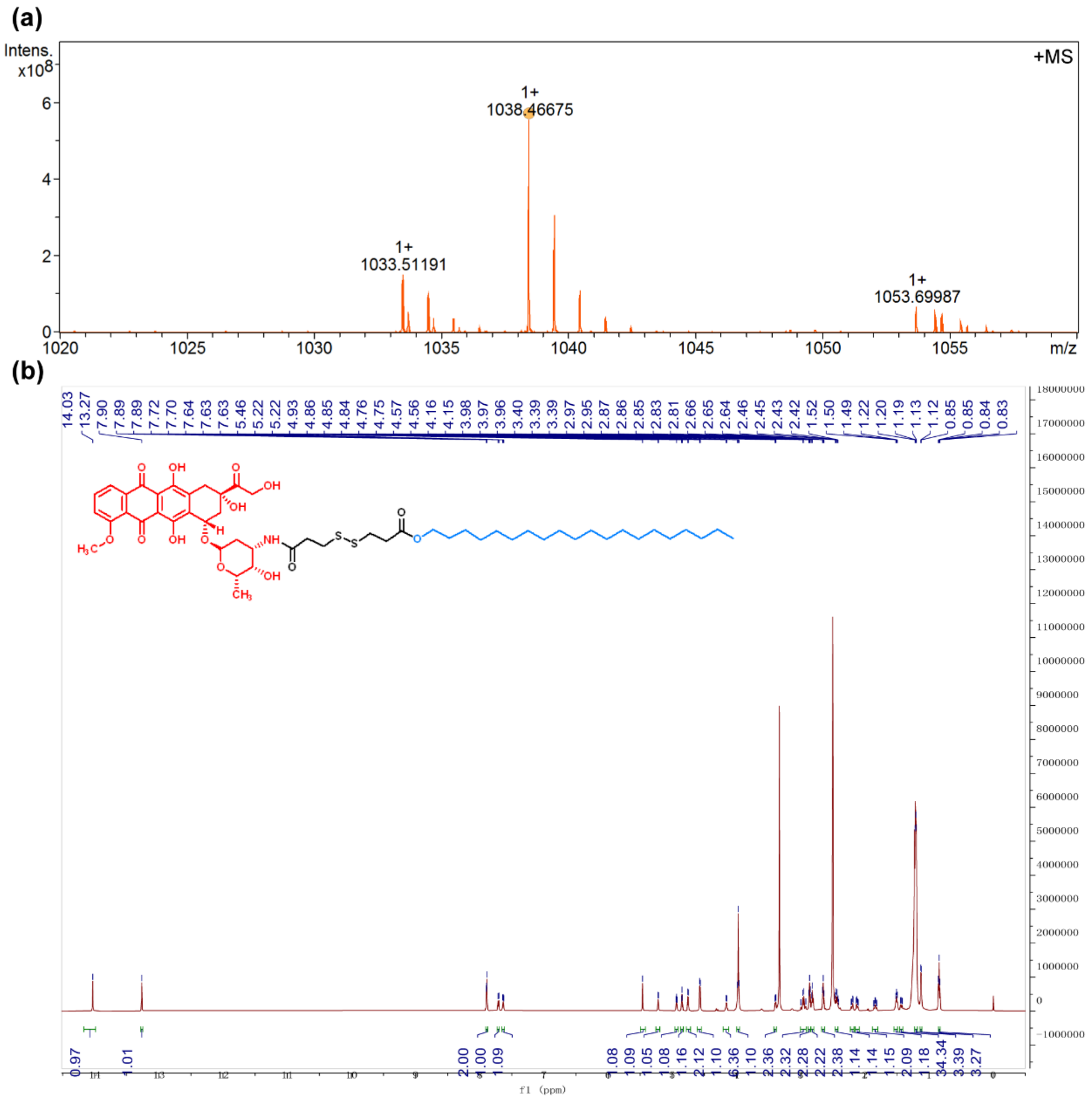

Figure S5. (**a**) MS of DSSC20 prodrug. $[M+Na]^+ = 1038.46675$. (**b**) ^{1}H NMR of DSSC20 prodrug. ^{1}H NMR (600 MHz, DMSO-*d*$_6$) δ 14.03 (s, 1H), 13.27 (s, 1H), 7.92–7.87 (m, 2H), 7.71 (d, *J* = 8.2 Hz, 1H), 7.66–7.61 (m, 1H), 5.46 (s, 1H), 5.22 (d, *J* = 3.8 Hz, 1H), 4.93 (t, *J* = 4.4 Hz, 1H), 4.85 (t, *J* = 6.0 Hz, 1H), 4.75 (d, *J* = 5.9 Hz, 1H), 4.57 (d, *J* = 6.2 Hz, 2H), 4.16 (q, *J* = 6.6 Hz, 1H), 4.00 – 3.95 (m, 6H), 3.41–3.37 (m, 1H), 3.01–2.90 (m, 2H), 2.86 (t, *J* = 6.8 Hz, 2H), 2.81 (t, *J* = 7.2 Hz, 2H), 2.65 (t, *J* = 6.8 Hz, 2H), 2.49–2.39 (m, 2H), 2.20 (dd, *J* = 14.5, 3.4 Hz, 1H), 2.12 (dd, *J* = 14.4, 5.7 Hz, 1H), 1.84 (td, *J* = 13.0, 4.0 Hz, 1H), 1.50 (t, *J* = 7.1 Hz, 2H), 1.43 (dd, *J* = 12.2, 4.6 Hz, 1H), 1.24–1.18 (m, 34H), 1.12 (d, *J* = 6.6 Hz, 3H), 0.84 (t, *J* = 7.0 Hz, 3H). The yellow dots represent the molecular ion peaks of the target product.

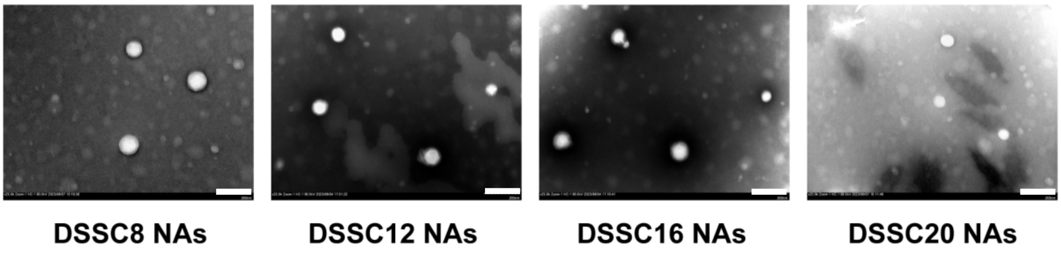

Figure S6. The transmission electron microscope image of DSSC8 NAs, DSSC12 NAs, DSSC16 NAs and DSSC20 NAs. Scale bar represents 200 nm.

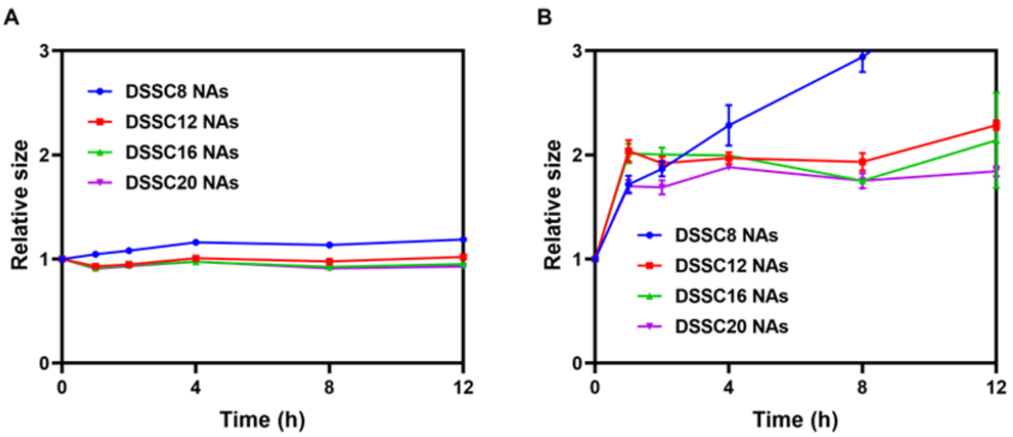

Figure S7. The size change of prodrug nanoassemblies in PBS without (**A**) or with (**B**) DTT (n = 3).

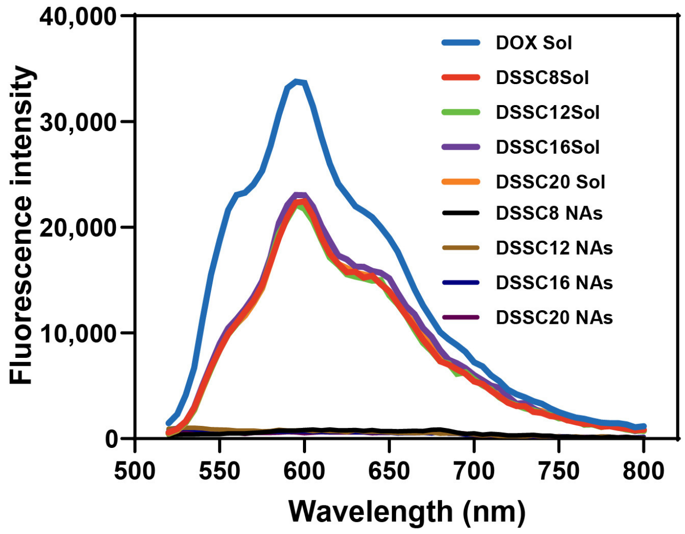

Figure S8. Fluorescence spectrum of DOX Sol, prodrug Sol and prodrug nanoassemblies at a DOX concentration of 2 μg/mL.

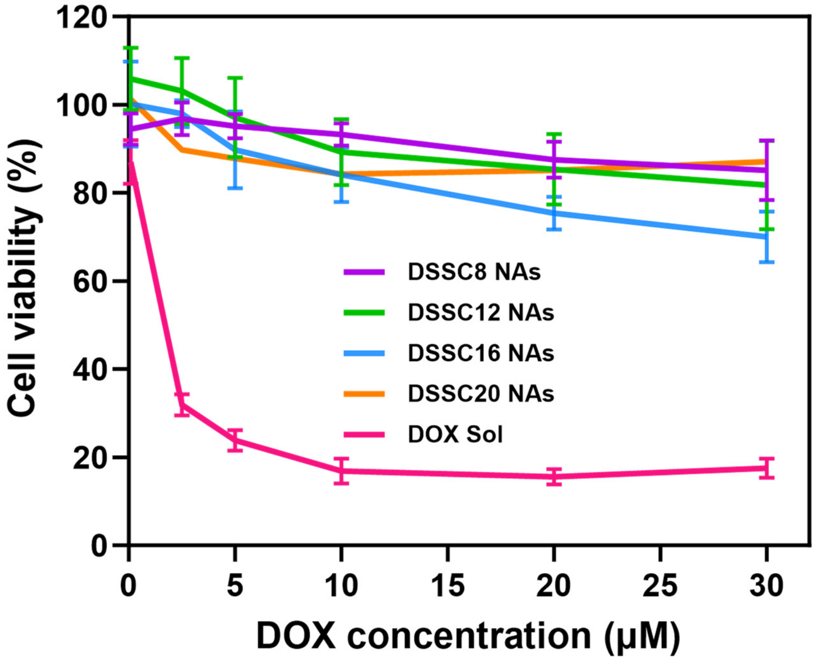

Figure S9. Cell viability after treated with various concentrations of DOX Sol and prodrug-nanoassemblies for 48 h in 3T3 cells.

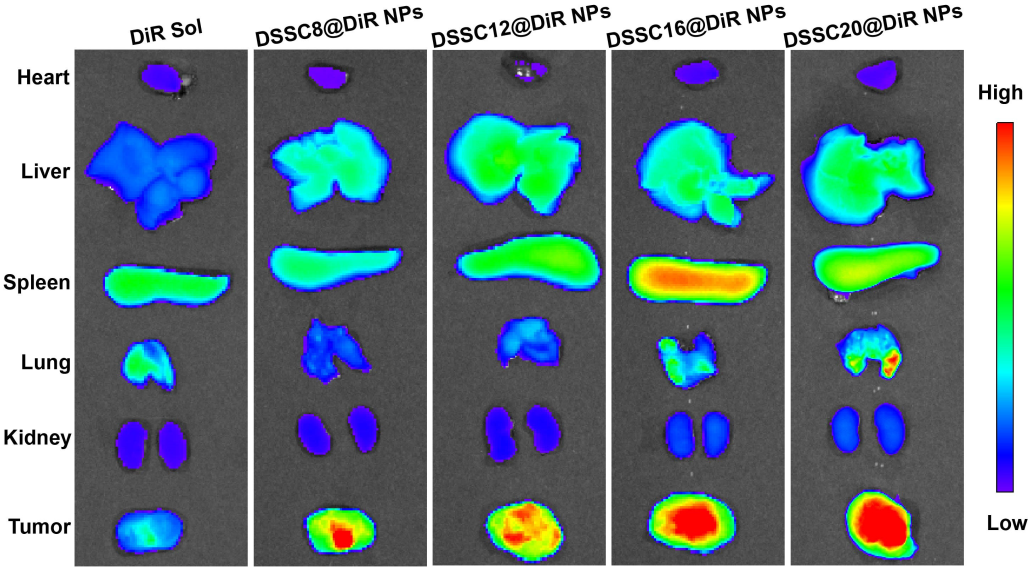

Figure S10. Ex vivo fluorescent imaging of 4T1 tumor-bearing BALB/c mice treated with DiR Sol and DiR-labeled prodrug-nanoassemblies at the time when tumor accumulation was brightest.

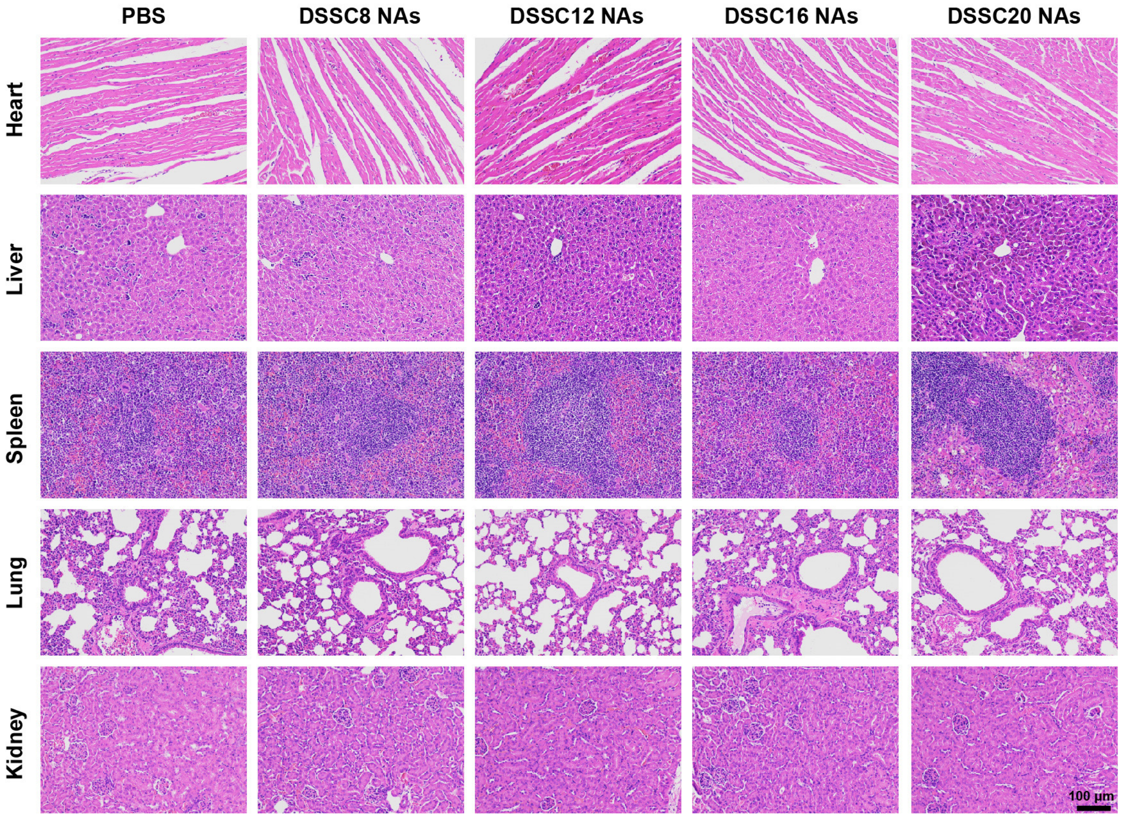

Figure S11. H&E staining images of the major organs of mice bearing 4T1 tumor xenografts after the last treatments (Scale bar = 100 μm).

Table S1. The characteristics of PEGylated prodrug-nanoassemblies (n = 3).

Formulations	Size (nm)	PDI	Zeta potential (mV)
DSSC8 NAs	107.2 ± 1.0	0.05 ± 0.04	−24.2 ± 2.0
DSSC12 NAs	77.2 ± 1.4	0.15 ± 0.05	−37.4 ± 0.9
DSSC16 NAs	76.6 ± 2.5	0.13 ± 0.08	−33.7 ± 0.7
DSSC20 NAs	66.4 ± 0.5	0.19 ± 0.03	−36.4 ± 0.7

Table S2. The characteristics of C6-labeled prodrug nanoassemblies (n = 3).

Formulations	Size (nm)	PDI	Zeta potential (mV)
DSSC8@C6 NAs	108.3 ± 1.8	0.11 ± 0.01	−34.3 ± 1.4
DSSC12@C6 NAs	92.8 ± 1.6	0.11 ± 0.05	−34.7 ± 1.0
DSSC16@C6 NAs	89.1 ± 3.8	0.23 ± 0.06	−30.6 ± 0.5
DSSC20@C6 NAs	83.8 ± 0.8	0.12 ± 0.05	−31.7 ± 1.2

Table S3. IC50 values (μmol/L) of DOX Sol and DOX prodrugs nanoassemblies against three tumor cell lines and 3T3 cell (n = 3).

Cell lines	DOX Sol	DSSC8 NAs	DSSC12 NAs	DSSC16 NAs	DSSC20 NAs
4T1	0.29 ± 0.01	53.05 ± 4.65	44.29 ± 2.05	15.78 ± 2.58	43.54 ± 4.82
RM-1	0.10 ± 0.01	126.00 ± 22.45	22.32 ± 1.61	12.38 ± 1.13	48.21 ± 7.40
CT 26	0.27 ± 0.05	29.22 ± 1.30	23.80 ± 0.89	15.46 ± 0.18	133.8 ± 34.68
3T3	1.33 ± 0.03	172.10 ± 82.30	149.50 ± 70.67	63.91 ± 11.33	127.20 ± 44.10

Data are presented as mean ± SD (n = 3).

Table S4. Pharmacokinetic parameters of DiR Sol and DiR-labeled prodrug nanoassemblies (n = 6).

Formulations	C_{2h} [a)]	$AUC_{0\text{–}36\ h}$ (μg/mL×h) [b)]
DiR Sol	0.85 ± 0.21	23.33 ± 4.61
DSSC8@DiR NAs	20.06 ± 1.63	196.46 ± 16.29
DSSC12@DiR NAs	24.93 ± 5.17	240.94 ± 52.36
DSSC16@DiR NAs	28.61 ± 1.89	332.49 ± 32.55
DSSC20@DiR NAs	29.63 ± 1.51	390.18 ± 24.19

[a)] The plasma concentration at 2 h; [b)] Area under the plasma concentration-time curve to infinity (μg/mL×h).
